# Why Not All the Powerful Abuse? The Competitive Effects of Psychological Distance and Self-Control

**DOI:** 10.3389/fpsyg.2021.730365

**Published:** 2021-09-09

**Authors:** Caiyun Huang, Siyu Tian

**Affiliations:** College of Business, Shanghai University of Finance and Economics, Shanghai, China

**Keywords:** social distance theory of power, abusive supervision, psychological distance, self-control, power, independent self-construal

## Abstract

Building on the social distance theory of power, this study proposed the positive and negative mechanisms of power and their impacts on abusive supervision from the competitive perspectives of psychological distance and self-control. The boundary effects of independent self-construal were also analyzed. The hypotheses of this study were tested through questionnaires and an experimental study design. The Study 1 data were collected from 422 supervisors and subordinates from five private enterprises and one state-owned enterprise in Eastern China. Study 2, on the other hand, was conducted through a scenario-based experiment in which 180 part-time master of business administration (MBA) students from a university in Eastern China participated. All data were tested using polynomial regression analysis and a bootstrapping appraisal. The results revealed that (1) the relationship between power and abusive supervision is not significant; (2) psychological distance mediates the relationship between power and abusive supervision, with high power leading to higher psychological distance, which, in turn, strengthens abusive supervision; (3) self-control mediates the relationship between power and abusive supervision, with high power leading to higher self-control, which, in turn, weakens abusive supervision; (4) the mediating effect of psychological distance is stronger, and the mediating effect of self-control is weaker when independent self-construal is high rather than low. At the end of this study, the theoretical and practical implications are discussed.

## Introduction

Over the past two decades, research into abusive supervision has been prolific. Abusive supervision, defined as “a subordinate's perception of the extent to which supervisors engage in the sustained display of hostile verbal and non-verbal behaviors, excluding physical contact” (Tepper, 2000, p. 178), is a prevalent and toxic phenomenon. Indeed, many studies have observed the deleterious effects abusive leadership has on a great number of individuals and on organizational outcomes (for reviews, see Martinko et al., 2013; Tepper et al., 2017; Yu et al., 2021). Although the negative consequences of abusive supervision are well-known, its antecedents received less attention from researchers (Martinko et al., 2013; Zhang and Bednall, 2016). In particular, Yu et al. (2021) found that, to date, only three articles have investigated the antecedents of abusive supervision in the hospitality industry. Thus, it is important to explore the nature of the existence of abusive supervision and understand how organizations can minimize or at least curb its occurrence.

The reasons leaders abuse their subordinates are mainly explored using two perspectives from the subordinate level (e.g., provocative behavior, anti-productive work behavior, high dependence, self-interest, passivity, and negative workplace gossip) (Tepper and Simon, 2015; Ye et al., 2021) and organizational level (e.g., poor relationships, negative organizational climates, and low organizational fairness) (Hoobler and Hu, 2013; Zhang and Bednall, 2016). However, researchers have recently turned their attention to investigating the antecedents of abusive supervision from supervisor-focused factors, such as supervisor traits or experiences (Peng et al., 2019). Among these studies, little research has suggested that the power advantage a leader has over their followers may trigger abusive supervision (Lam and Xu, 2019). However, it is undeniable that some leaders with power will adopt the strategy of abusing their subordinates (Ouyang et al., 2015; Zheng and Liu, 2017; Tu et al., 2018). Despite these observations, the relationship between the power of a supervisor and their abusive supervision has not yet been verified (Wee et al., 2017) through the possible reasons for the complexity of a power functioning system. Thus, how power affects the abusive behaviors of leaders is one of the issues that this study aimed to address.

Based on the social distance theory of power, this study proposed that the power of leaders influences their tendencies to abuse subordinates mainly through two mechanisms. First, the holders of power will have more psychological distance than those who are powerless (Magee and Smith, 2013), which may trigger abusive supervision. With this, the powerful are not inclined to make contact with the powerless, which weaken the empathy of the powerful leaders and projection on others; their concern for the needs and psychological state of others will also be reduced. Thus, the powerful are more likely to engage in abusive behaviors. Second, powerholders will construe goals at a higher level, further inducing self-control. Self-control is exercised by suppressing automated processing and impulsive responses according to long-term goals (Trope and Fishbach, 2000). When problems arise, the powerful tend to forgive others to achieve their goals, thereby easing the tension in relations (Guinote, 2007). This type of behavior leads to a reduction in the occurrence of abusive supervision.

The examination of the coexistence of the two mechanisms discussed is essential to understanding why a leader with power is likely to act in an abusive manner. Meanwhile, an equally important question is what factors will affect the way leaders perceive and use their power. Furthermore, this study examined how the independent self-construal of leaders qualifies their reactions to power. In particular, independent self-construal means the tendency to differentiate between oneself and others (Singelis, 1994). After all, power and abusive supervision are essentially interpersonal and relational. Specifically, this study argues that powerful people with high independent self-construal will experience more psychological distance and less self-control, further influencing their abusive supervision.

This study makes several theoretical contributions. First, this research complemented and extended abusive supervision research and provided a strong theoretical framework. This was achieved by studying the relationship between power and abusive supervision from the initial mechanisms at the level of the supervisor. Furthermore, prior studies have primarily explored the antecedents of abusive supervision from the organization and subordinate levels, while little research from the supervisor-level perspective has been conducted. In addition, little direct empirical evidence exists, which demonstrates the relationship between power and abusive supervision (e.g., Eissa and Lester, 2017; Khan et al., 2017; Wee et al., 2017). Furthermore, Mooijman et al. (2015) proposed that power is the fundamental reason why leaders inflict punishment, because power increases the reliance on the deterrence mediated by distrust. Based on the social distance theory of power, this study attempted to untie the black box of the mechanism between power and abusive supervision and to supplement the research about abusive supervision at the level of the supervisor.

Second, our research advanced the existing research on the uncertain relationship between power and abusive supervision by exploring the mediation of psychological distance and self-control from two opposite aspects. While most studies have focused on different moderators or mediators to explain why all leaders do not abuse their subordinates or why all these subordinates are not abused (e.g., Tepper and Simon, 2015; Courtright et al., 2016; Zhang and Bednall, 2016; Khan et al., 2017; Tepper et al., 2017; Wee et al., 2017), very few of them paid any attention to the opposite effects of power. When applying the social distance theory of power, this study came up with a detailed explanation of how power influences abusive supervision. Specifically, this study employed psychological distance and self-control as a bridge that links power and abusive supervision, respectively. In addition, this study further expanded the adaptability of the theory to different contexts.

Finally, this study further explored the boundary conditions under which abusive supervision is more or less enhanced by the power leaders have. Some studies have confirmed that not all leaders will abuse subordinates, given the moderation of the traits of the supervisor (e.g., the mindfulness of the supervisor, neuroticism, conscientiousness, agreeableness, gender, and situation-control) (Courtright et al., 2016; Liang et al., 2016; Eissa and Lester, 2017; Khan et al., 2017), the traits of their subordinates (e.g., the capacity a subordinate has for self-control, turnover intentions, and negative affectivity) (Aquino and Bradfield, 2000; Lian et al., 2014), and the characteristics of the organization(e.g., leader-member exchange and downsizing) (Martinko et al., 2011; Neves, 2014). However, few researchers have noticed the moderator from the perspective of interpersonal aspects. This study identified the independent self-construal level of leaders as a moderator, mainly considering their attention from themselves to others. Furthermore, this research endeavor highlighted the importance of considering the personalities of leader when their power exerts effects on their abusive supervision. The study also offered a contingency perspective for understanding the relationships between power and abusive supervision. The discussion of boundary conditions delineated clearer conditions for the main effect of the power of leaders.

## Theory and Hypotheses

### Power and Abusive Supervision

To date, a small body of literature has explored the relationship between power and abusive supervision. Power in this study is defined as asymmetric control over valued resources (Depret and Fiske, 1993). On the other hand, leaders, as powerholders, can influence subordinates by allocating resources or administering punishment (Lian et al., 2014). In particular, some researchers have implied that the power advantage leaders have over subordinates may trigger abusive supervision (e.g., Tepper et al., 2009, 2015; Lam and Xu, 2019). Mooijman et al. (2015) also further proposed that power is the fundamental reason why leaders inflict punishment, since power increases the reliance on the deterrence mediated by distrust. Although power asymmetry can give managers ample opportunities to abuse their subordinates (Aryee et al., 2007), a meta-analysis study found that the effects of the power and abusive supervision of supervisors were not significant (Zhang and Bednall, 2016). Therefore, it seems that little research has been conducted to specifically explore how power affects abusive behavior thus far.

This study, based on the social distance theory of power, attempted to explore the relationship between power and abusive supervision from the perspectives of the competitive mechanisms of power. Specifically, this theory puts forward two basic principles (Magee and Smith, 2013). One is that asymmetric dependence between two individuals leads to the asymmetric experiences of social distance, with the powerful consequently feeling more subjective distance than the powerless. The second principle is that a greater sense of social distance leads the powerful party to engage in more abstract mental representation (i.e., higher-level construal) than the weak party. The former causes the powerful to abuse more; the latter causes them to abuse less. Therefore, we hypothesize the following:

Hypothesis 1: The power of leaders is not related to abusive supervision.

### Psychological Distance as a Mediator

According to the social distance theory of power, power makes individuals experience different degrees of social distance; for instance, powerholders will perceive more social distance (Magee and Smith, 2013). Social distance mainly depends on two factors: (1) the motivation to establish connections with others and (2) the expectation that others will have the motivation to establish connections with ourselves (Magee and Smith, 2013). Social distance can be thought of as a form of psychological distance, defined as “a subjective experience that something is close or far away from the self, here, and now” (Trope and Liberman, 2010, p. 440; Magee and Smith, 2013). Specifically, psychological distance is comprised of four forms: temporal, spatial, hypothetical, and social (Trope and Liberman, 2010). In an interpersonal relationship, psychological distance is mainly reflected in the social distance, which is, in turn, is mostly related to power (Magee and Smith, 2013).

Having power also means having more resources, influence, control, and freedom (Galinsky et al., 2003). With this, the powerful are less dependent on others for goal satisfaction. As such, the powerful can act arbitrarily, without being influenced by others (Guinote, 2007). On the contrary, the powerless usually need to rely on the resources of other people. Thus, the powerless have a stronger willingness to establish contact with others (van Kleef et al., 2008), and the powerful have a lower motivation to establish connections with others. Moreover, those with power tend to interpret the motivation of others to establish contact with them as purposeful, which further increases their psychological distance. For instance, Inesi et al. (2012) confirmed that powerholders with high levels of power are apt for making cynical attributions about the intentions of low-power affiliation attempts become closer when they realize that these powerholders with high power possess resources of value. In addition, the social class structure has also been found to further widen the psychological distance perceived by powerful individuals (Lammers et al., 2012).

Furthermore, the high psychological distance induced by power further causes the powerful to engage in more abusive behaviors toward the powerless (Wee et al., 2017). When individuals have high psychological distances (compared with the interests of others), they pay more attention to their own interests (Zhang et al., 2018; Paramita et al., 2020) and act more consistently with their preferences and goals (Galinsky et al., 2008). They will also be less concerned about others' views (Galinsky et al., 2006) and may even materialize others (Overbeck and Park, 2001), inducing less concern about others' feelings, emotions and goals. Additionally, these individuals only care about their goals being achieved, often ignoring whether their words and deeds bring discomfort to others. Consequently, the probability of showing adverse behaviors to others, such as abusive supervision toward subordinates, may even be increased. Therefore, the following hypothesis was proposed:

Hypothesis 2: Psychological distance mediates the relationship between the power of leaders and their abusive supervision.

### Self-Control as a Mediator

The social distance theory of power further proposes that powerholders with a greater sense of social distance tend to engage in the higher-level construal of targets with higher self-control ability (Magee and Smith, 2013). Specifically, the powerful are likely to make decisions at higher levels, thus paying more attention to the final results of decisions. They also do not care about the details of the decision-making process. Furthermore, in this process, the powerful tend to control the automatic thinking and impulsive reactions caused by short-term goals (Trope and Fishbach, 2000). However, the cognitive flexibility and cognitive selectivity of these powerholders show that they can, if necessary, better control themselves to ensure the achievement of goals (Magee and Smith, 2013). Studies have also further confirmed that a sense of power will lead to higher self-control. For example, people with high power have less impulsive buying behaviors (Rucker et al., 2012), as these individuals would rather save more money than have fun at the moment (Garbinsky et al., 2014). In other words, they can sacrifice the present demands for greater rewards in the future (Joshi and Fast, 2013).

Powerholders with high self-control will restrain their impulsive behaviors, including behaviors such as abusive supervision. In particular, high self-control represents the efforts of an individual toward achieving long-term goals. Correspondingly, they will not be easily interfered with by other events and have strong regulatory control abilities (Liang et al., 2016). Thus, such people have higher perception thresholds when facing negative information, consequently becoming less likely to be affected by the outside world. For example, Chen et al. (2019) found that athletes with high self-control could adapt to their living environments better and show less aggressive behaviors. The reverse was true for individuals with low self-control, who caused more abusive supervision (Courtright et al., 2016; Liang et al., 2016). Furthermore, Liang et al. (2016) also found that abusive supervision represents a failure to exhibit self-control when facing provocation. Barnes et al. (2015), in addition, suggested that insufficient self-control is caused by poor sleep at night that, in turn, leads to more abusive supervision. Therefore, to achieve their long-term goals, individuals with high self-control strive not to be affected by interference factors; they ensure their words and deeds conform to the norms and show low levels of abusive behavior. Therefore, Hypothesis 3 was proposed:

Hypothesis 3: Self-control mediates the relationship between the power of leaders and their abusive supervision.

### Independent Self-Construal as a Boundary Condition

This study proposed that leaders respond to their power using abusive supervision through psychological distance or self-control mechanisms depending on their level of independent self-construal. Independent self-construal is defined as “a bounded, unitary, stable self that is separate from social context” (Singelis, 1994, p. 581). As such, individuals with high independent self-construals tend to think in terms of “me” and have their abilities, attributes, characteristics, interests, or goals as referents rather than those of others (Singelis, 1994; Holland et al., 2004; Utz, 2004).

People with independent self-construals also tend to maintain longer interpersonal distances and are more inclined to consider their own self-interests. As previously mentioned, the powerful are more likely to perceive psychological distances than the powerless. This happens because independent self-construals can activate the contrast effect; thus, people also tend to find what makes them unique and widen the distance between themselves and others (Stapel and Koomen, 2001). Furthermore, they also become less likely to embed themselves within the scopes of others (Markus and Kitayama, 1991) or take the perspective of others into account (Wu et al., 2019). When powerholders simultaneously have higher independent self-construals, they become less likely to consider the thoughts and feelings of their subordinates (Utz, 2004). Instead, these people tend to prefer to use their power to keep away from others, in case they might interrupt the achievement of the goals of the powerholders themselves. Thus, independent self-construals enlarge the psychological distance brought by high power. Therefore, Hypothesis 4 was proposed:

Hypothesis 4: Independent self-construals moderate the relationship between the power of leaders and their psychological distances such that the relationship becomes stronger under high (rather than low) independent self-construals.

Meanwhile, high power brings about a higher level of self-control, which, in turn, helps to ensure the harmony of interpersonal relationships while not interfering with the achievement of the goals of an individual (Guinote, 2007). As previously mentioned, individuals with independent self-construals pay more attention to their achievements and autonomy and actively seek various opportunities. These individuals show a focus on “promotion” (Lee et al., 2000). When the self-construals of leaders tend to be independent, the individuals also have greater consideration for themselves and focus more on internal information than on long-term goals. Additionally, these people focus more on their own abilities and feelings, showing more selfishness and willfulness (Howard et al., 2007) that, in turn, make them more likely to do things that undermine the interests of others (Guinote, 2007). This means that independent self-construal causes the powerful to act arbitrarily and recklessly; they are less likely to control their behaviors to achieve long-term goals. Thus, independent self-construal reduces the self-control brought about by high power. As such, Hypothesis 5 was proposed:

Hypothesis 5: Independent self-construals moderate the relationship between the power of leaders and their self-control in such a way that the relationship is weaker under high (rather than low) independent self-construals.

Based on the previous argument, this study further proposed that independent self-construals moderate the mediating role of psychological distance and self-control in the relationship between the power of leaders and their abusive supervision. For instance, when the self-construal of an individual tends to be independent, the power of leaders also has more indirect positive impacts on abusive supervision through the mediation of psychological distance. Conversely, leaders have less intense indirect negative impacts on abusive supervision through the mediating role of self-control. To be specific, independent self-construals cause the relationship between the power of leaders and their psychological distances to be stronger, thereby increasing the level of abusive supervision. In the same way, such construals also cause the relationship between the power of leaders and their self-control to become weaker, thereby increasing the levels of abusive supervision. Thus, the following hypotheses were proposed:

Hypothesis 6: Independent self-construals moderate the indirect effect of the power of leaders on abusive supervision *via* psychological distance in such a way that this indirect effect is stronger under the influence of high (rather than low) independent self-construals.
Hypothesis 7: Independent self-construals moderate the indirect effect of the power of leaders on abusive supervision *via* self-control in such a way that this indirect effect is weaker under the influence of high (rather than low) independent self-construals.

## The Current Study

**Figure 1:**
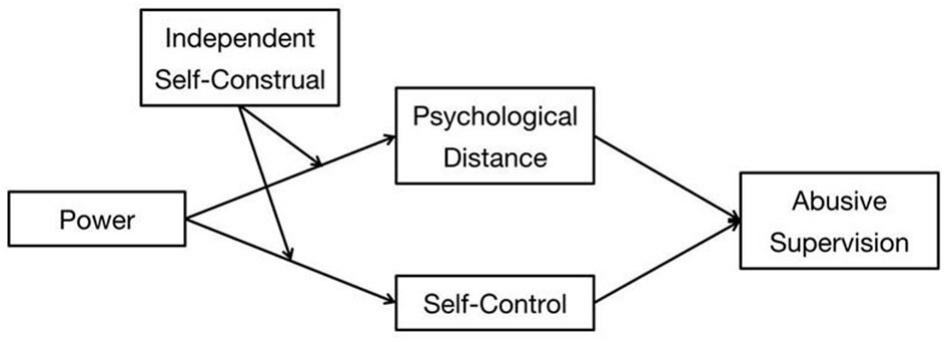
Hypothesized model.

To test the theoretical model depicted in Figure 1, a field study was conducted (Study 1), followed by a scenario experiment (Study 2). In Study 1, supervisors were asked to rate their power, psychological distance, self-control, and independent self-construal; employees were asked to rate the abusive supervision of their supervisors. While the field study provided robust evidence for the external validity of this core effect, a scenario experiment was employed in Study 2 to establish the internal validity of the model used in this study. Considered together, these two studies comprised a mix of different designs and samples that provided a good combination of both internal and external validity evidence for the theoretical model.

### Study 1 Method

#### Sample and Procedure

The sample comprised leader–follower dyads from five private enterprises and one state-owned enterprise in East China. The study was conducted in 2020 with two sets of questionnaires: one for leaders and the other for their immediate subordinates. The questionnaires were anonymously packed in two envelopes. One envelope contained the leader questionnaire that was used to measure power, psychological distance, self-control, and self-construal, while the other envelope contained the subordinate questionnaire that was used to measure abusive supervision. The researcher also contacted the human resources (HR) managers in advance and confirmed the leader–subordinate matching list and sites. Before the questionnaires were distributed, the researchers coded the questionnaires for matching purposes. The questionnaires were then distributed on-site, and the employees who were absent or otherwise unable to fill in the questionnaire were immediately eliminated. After each participant completed a questionnaire, they put the finished questionnaire back in the envelope and handed it to the researcher. In all, 512 subordinates and 499 leaders responded, and 461 matched samples were obtained. After excluding unusable cases and matching leader and subordinate reports, a total of 422 valid questionnaires remained, with an effective rate of 91.5%. Of the supervisor respondents, 68.5% were men, with most being relatively young (43.6% were from 30 to 40 years of age) and well-educated (75.8% held 3-year college degrees or above). Of the subordinate respondents, 50.7% were men, with most being young (63.3% were from 18 to 30 years of age) and well-educated (79.1% held 3-year college degrees or above).

#### Measures

The measurement inventories were translated and back-translated (Brislin, 1970) to assure their appropriateness in Chinese. Unless otherwise noted, all responses were rated on a five-point Likert scale (from 1 = “strongly disagree” to 5 = “strongly agree”).

##### Leaders' Power

Leaders' power was assessed using an eight-item scale developed by Anderson and Galinsky (2006). One sample statement was “I think I have a great deal of power”. Cronbach's alpha for the scale was 0.81.

##### Self-Control

Supervisors rated a 13-item scale developed by Tangney et al. (2004). One sample statement was “I refuse things that are bad for me”. Cronbach's alpha for the scale was 0.86.

##### Psychological Distance

This variable was measured with a seven-item scale adopted from Salzmann and Grasha (1991). One sample statement was “I am very patient with others” (α = 0.97). The original scale consisted of 11 items. In order to avoid understanding difficulties caused by language differences, the scale was simplified and reduced to seven items after interviewing experts and employees.

##### Independent Self-Construal

This variable was measured using a three-item scale adopted from Singelis (1994) (α = 0.82). One sample statement was “My personal identity, independent of others, is very important to me”.

##### Abusive Supervision

Employees rated abusive supervision by adopting a five-item scale by Mitchell and Ambrose (2007) (α = 0.79), which was a shortened version of the original 15-item scale developed by Tepper (2000). A sample statement was “My supervisor tells me my thoughts or feelings are stupid”.

##### Control Variables

The authors controlled for the gender, age, and tenure of both the leaders and employees.

### Study 1 Results and Discussion

The descriptive statistics and correlations for all the study variables are reported in Table 1. All the control variables (gender, age, and tenure of the employees and leaders) were not related to abusive supervision in this study. Psychological distance was related to the gender (*r* = 0.1, *p* < 0.05) and age (*r* = 0.1, *p* < 0.05) of the employees and the age (*r* = 0.17, *p* < 0.01) of the leaders. Self-control was related to the gender (*r* = 0.1, *p* < 0.05) and tenure (*r* = 0.16, *p* < 0.01) of the employees and the tenure (*r* = 0.12, *p* < 0.05) of the leaders.

**Table 1:** Descriptive statistics and correlations of Study 1.

**Variable**	***Mean***	***SD***	**1**	**2**	**3**	**4**	**5**	**6**	**7**	**8**	**9**	**10**	**11**
1. Employee gender	1.49	0.50											
2. Employee age	3.08	0.95	−0.06										
3. Employee tenure	6.40	4.89	−0.02	0.17^***^									
4. Leader gender	1.32	0.47	−0.09	0.01	−0.03								
5. Leader age	3.83	1.31	−0.07	0.10^*^	0.11^*^	0.04							
6. Leader tenure	13.19	7.94	0.02	0.07	0.01	−0.06	0.21^***^						
7.Leaders' power (LP)	3.44	0.64	0.03	0.09	0.06	0.06	0.04	−0.09	**(0.81)**				
8. Psychological distance	3.48	1.19	0.10^*^	0.10^*^	0.09	0.01	0.17^**^	0.06	0.23^***^	**(0.86)**			
9. Self–control	3.63	0.53	0.10^*^	−0.02	0.16^**^	0.08	−0.09	0.12^*^	0.10^*^	0.22^***^	**(0.97)**		
10. Independent self–construal (IND_SC)	3.03	0.80	−0.07	−0.07	−0.13^**^	0.02	−0.03	0.05	−0.04	−0.50^***^	−0.01	**(0.82)**	
11. Abusive supervision	2.22	0.70	0.01	0.00	0.05	−0.03	0.06	0.03	−0.07	−0.21^***^	0.16^**^	0.06	**(0.79)**

*N = 422. Bold value means reliability estimates (coefficient alpha) are on the diagonal*;

* *p < 0.05*,

** *p < 0.01, and*

*** *p < 0.001*.

A confirmatory factor analysis (CFA) was also conducted to examine any distinction of the main variables included in the study. A five-factor model was found to have a good fit (χ^2^/df = 2.41; CFI = 0.94, TLI = 0.93, RMSEA = 0.06, SRMR = 0.04), which was also better than the fits of alternative models when power was combined with two mediators (χ^2^/df = 7.64; CFI = 0.72, TLI = 0.69, RMSEA = 0.13, SRMR = 0.14) and all variables (χ^2^/df = 19.8; CFI = 0.18, TLI = 0.12, RMSEA = 0.21, SRMR = 0.25). These results provided support for the discriminant validity of the measures used in this study. Thus, all the variables were treated as separate variables in the subsequent analyses performed in this study.

#### Hypotheses Testing

Table 2 summarizes the regression results. Hypothesis 1 stated that the power of leaders is not related to abusive supervision; the results also showed that power was not related to abusive supervision (M5: b = −0.08, *n.s*.). Thus, Hypothesis 1 was supported. Hypothesis 2 assumed that the power of leaders has an indirect effect on abusive supervision through psychological distance. The results indicated that power was positively associated with psychological distance (M1: *b* = 0.39, *p* < 0.001), while M6 showed that psychological distance was positively associated with abusive supervision (*b* = 0.13, *p* < 0.001). Hence, psychological distance played a mediating role in the relationship between the power of leaders and their abusive supervision [indirect effect *b* = 0.07, 95% CI = (0.02, 0.14), with a 95% CI that does not contain 0 meaning that the result is significant; the same below]. Thus, Hypothesis 2 was supported. In addition, power was positively related to self-control (M3: *b* = 0.23, *p* < 0.001), while self-control was negatively related to abusive supervision (M6: *b* = −0.34, *p* < 0.001). In line with the expectations of this study, power had a negative indirect effect on abusive supervision *via* self-control [indirect effect *b* = −0.06, 95% CI = (−0.17, −0.01)]. Thus, Hypothesis 3 was supported.

**Table 2:** Regression results of Study 1.

**Variable**	**Psychological distance**		**Self–control**		**Abusive supervision**			
	**M1**	**M2**	**M3**	**M4**	**M5**	**M6**	**M7**	**M8**
**Intercept**	0.84	3.45	2.62	2.55	2.36	3.15	2.21	2.45
Employee gender	0.26^*^	0.15	−0.03	−0.02	0.02	−0.03	0.01	−0.02
Employee age	0.07	0.03	0.07	0.07^**^	−0.00	0.01	−0.01	0.01
Employee tenure	0.01	0.00	0.00	0.00	0.01	0.01	0.01	0.01
Leader gender	0.00	0.02	−0.12^*^	−0.12^*^	−0.04	−0.07	−0.04	−0.08
Leader age	0.13^**^	0.11^**^	0.03	0.04	0.03	0.02	0.03	0.02
Leader tenure	0.01	0.01	0.01^*^	0.01^*^	0.00	0.00	0.00	0.00
LP	0.39^***^	0.41^***^	0.23^***^	0.21^***^	−0.08	−0.05	−0.06	−0.06
IND_SC		−0.76^***^		0.03			0.03	0.18^***^
LP^*^IND_SC		0.35^***^		−0.16^**^			−0.23^**^	0.11
Psychological distance						0.13^***^		0.19^***^
Self–control						−0.34^***^		−0.35^***^
*R^2^*	0.10	0.34	0.13	0.15	0.01	0.10	0.04	0.14
Δ*R*^2^	–	0.24^***^	–	0.02^**^	–	0.09^***^	0.03^**^	0.10^***^
*F*	6.33^***^	23.51^***^	8.46^***^	7.84^***^	0.74	5.08^***^	1.94^*^	6.25^***^

*N = 422*;

* *p < 0.05*,

** *p < 0.01, and*

*** *p < 0.001*.

**Figure 2:**
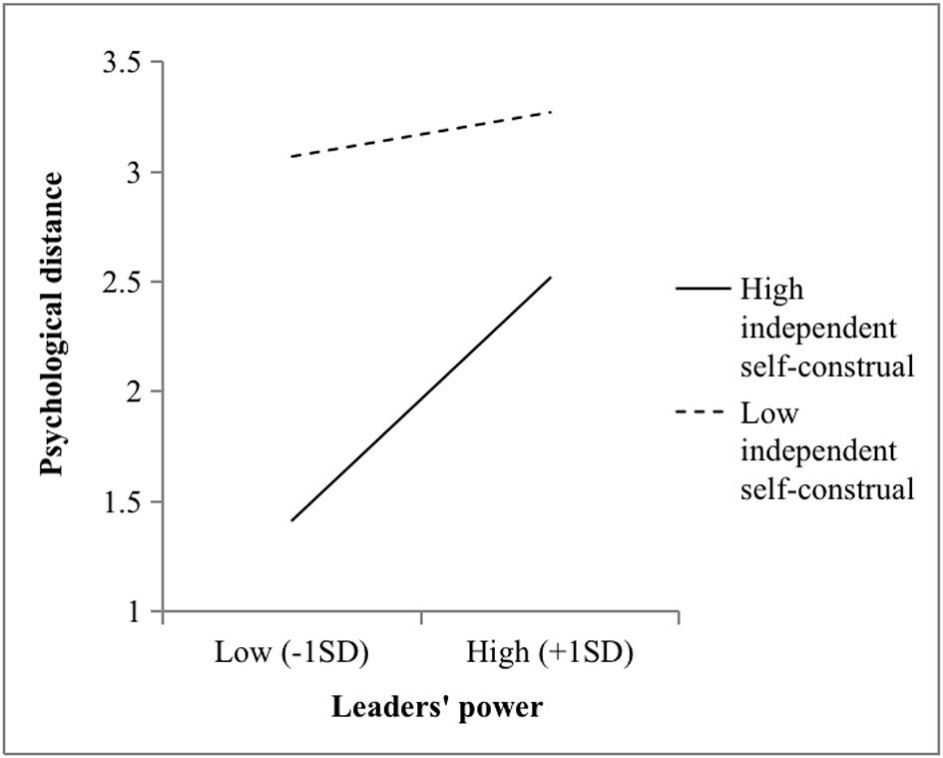
Two-way interaction effect between the power and independent self-construals of the leaders on psychological distance in Study 1.

**Figure 3:**
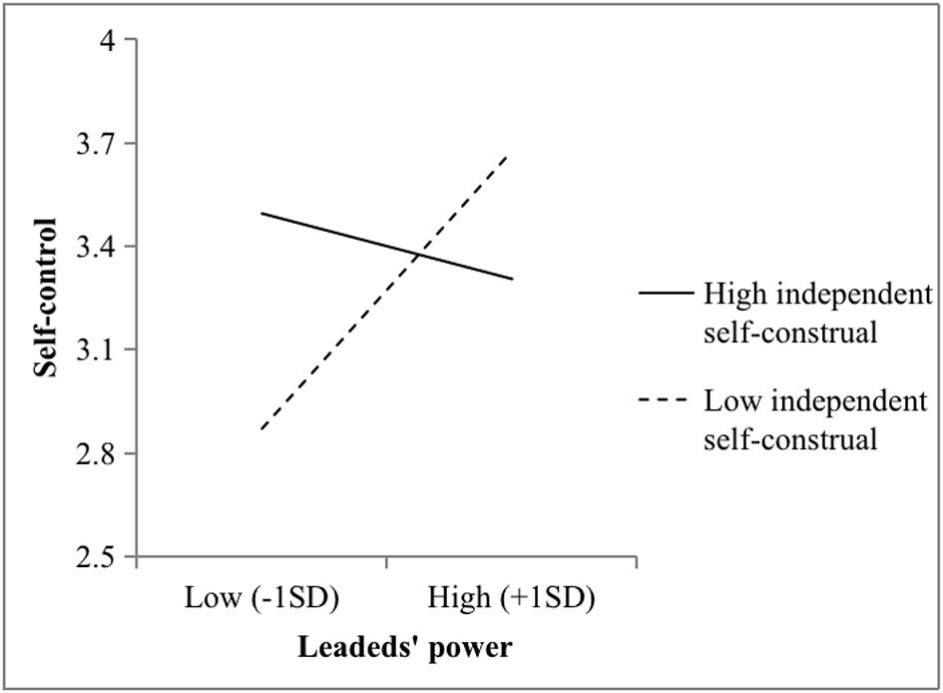
Two-way interaction effect between the power and independent self-construals of the leaders on self-control in Study 1.

Hypotheses 4 and 5 stated that independent self-construals moderate the relationship between power and psychological distance and between power and self-control, respectively. In this study, M2 showed that power and independent self-construal interacted to predict psychological distance (*b* = 0.35, *p* < 0.001). As illustrated in Figure 2, the power of leaders strongly fostered psychological distance among the supervisors with higher independent self-construals (*b* = 0.81, *p* < 0.001), while the relationship was not significant when the independent self-construals of the supervisors were lower (*b* = 0.39, *n.s*.). Again, there was a two-way interaction effect (M4: *b* = −0.16, *p* < 0.01) on self-control. Furthermore, Figure 3 illustrates that the positive effects of power and self-control were stronger among the supervisors with lower independent self-construals (*b* = 0.39, *p* < 0.01). Conversely, the relationship was not significant when the independent self-construals of the supervisors were higher (*b* = −0.07, *n.s*.). Hypotheses 4 and 5 were thus supported.

To test the moderated mediation relationships posited in Hypotheses 6 and 7, the moderated path analysis approach was applied to estimate two sets of effects at the high and low levels of the moderator (Edwards and Lambert, 2007). The results showed that the indirect effect *via* psychological distance was more pronounced when the independent self-construals of the supervisors were high [*b* = 0.11, *p* < 0.001, 95% CI = (0.06, 0.19)] rather than low [*b* = 0.82, *n.s*., 95% CI = (−0.01, 0.06)]. Additionally, the difference between these two indirect effects was also significant in this study [*b* = 0.09, *p* < 0.01, 95% CI = (0.04, 0.16)]. Thus, this finding means that independent self-construals moderated the indirect relationship between the power of leaders and their supervision *via* psychological distance, thereby supporting Hypothesis 6. Again, the indirect effect *via* self-control was more pronounced in leaders that had low independent self-construals [*b* = −0.08, *p* < 0.01, 95% CI = (−0.20, −0.04)] than when they had high independent self-construals [*b* = −0.02, *n.s*., 95% CI = (−0.01,0.11)]. Additionally, the difference between these two indirect effects was also significant [*b* = 0.06, *p* < 0.05, 95% CI = (0, 0.16)], meaning that independent self-construals moderated the indirect relationship between the power of leaders and their supervision *via* self-control. Thus, Hypothesis 7 was supported.

#### Discussion

The results from the field study provided initial support for the hypothesized model in this study. Psychological distance and self-control were found to mediate the relationship between the power of leaders and their abusive supervision, respectively. Furthermore, the different levels of independent self-construal were confirmed as the boundary condition. However, independent self-construal and psychological distance were measured with items adopted from the original 12- and 11-item scales, respectively, and whether the adopted items fully measured the two variables remains in doubt. Although this study provided strong external validity for the theoretical model, its conclusion needed constructive replication. Thus, the following scenario-based study was conducted to increase the internal validity of our conclusions and rule out alternative explanations for the findings of this study.

### Study 2 Method

#### Sample and Procedure

Drawing on previous manipulations of mental role-play (Rucker et al., 2012) in power research, a scenario-based experiment was conducted to replicate the findings of this study. A total of 197 part-time master of business administration (MBA) students were recruited from a university in Eastern China. All these MBA students had work experience, and most of them were leaders in their organizations; thus, they had proper experiences of abusive supervision and could better understand the manipulation of the study. Participation in the study was voluntary. After excluding subjects who did not meet the quality control questions, 180 valid samples remained. Among them, 62.7% were men, with most participants (78.9%) aged between 26 and 35 years; tenure was mainly (87.2%) from 1 to 10 years.

Participants were randomly assigned to either the leader with high power or the leader with low power. Each participant was presented with the scenario experiment through paper-and-pencil questionnaires. Participants were first assessed on several individual differences and then read one of two scenarios. Both scenarios instructed participants to imagine that they were the manager supervising four or five employees, with themselves being supervised by a director manager in a large company designing and manufacturing mobile phones. In both scenarios, participants were presented with a set of two situations. Following the scenarios, participants completed manipulation checks and measures of the power of the leader and their self-control, psychological distance, and abusive supervision.

#### Manipulations

Participants were asked to imagine themselves to be a research and development (R&D) department manager in a large company. In the condition where the leader had high power, they read the following manipulation:

“*In the daily work, I can get my department members to listen to what I say, and I never worry that my ideas will be questioned. Specifically, I can directly make decisions on many things, such as the R&D process and the specific work content of those processes, and I can formulate evaluation criteria. I can also get each member to do what I want. In addition, I would evaluate everyone according to the evaluation criteria every month, but they cannot know my evaluation of themselves, and they even have no chance to evaluate me”*.

In the condition where the leader had low power, they read the following manipulation:

“*In the daily work, I cannot get my department members to listen to what I say, and I often worry that my ideas will be questioned. Specifically, there are many things I need to make decisions about with members, such as the R&D process and the specific work content, as well as formulating evaluation criteria. However, my ideas are often ignored by the team members, and eventually, they will complete the work according to their own ideas. In addition, I would evaluate everyone according to the evaluation criteria every month. Sometimes, they will know my evaluation of themselves, and they even have a chance to evaluate me”*.

#### Measures

Unless noted otherwise, all the measures adopted a five-point Likert scale (1 = strongly disagree, 5 = strongly agree).

##### Leaders' Power

The same eight-item measure from Study 1 was used to check the manipulation of the power of leaders (α = 0.96).

##### Self-control

The same scale was adopted as in Study 1 (α = 0.9).

##### Psychological Distance

This variable was measured with an 11-item scale adopted from Salzmann and Grasha (1991). A sample statement was “I am very patient with others” (α = 0.96).

##### Independent Self-construal

This variable was measured with a 12-item scale adopted from Singelis (1994). One sample statement was “My personal identity, independent of others, is very important to me” (*α* = 0.7).

##### Abusive Supervision

The same scale was adopted as in Study 1 (α = 0.82).

### Study 2 Results and Discussion

#### Manipulation Check

Before testing the hypotheses, a manipulation check for power as a between-subject factor was performed. Participants were asked how strongly they agreed with an eight-item measure of the power of the leaders. Participants in the high-power condition (*M* = 4.19, *SD* = 0.39) rated this measure higher than those in the low-power condition (*M* = 2.14, *SD* = 0.51), *F*_(1, 178)_ = 30.19, *p* < 0.001. The results provided strong evidence for the efficacy and validity of the manipulation featured in the scenarios of this study.

#### Descriptive Statistics and Correlations

Table 3 reports the descriptive statistics and zero-order correlations among the variables. None of the control variables (gender, age, and tenure) were related to abusive supervision and/or psychological distance. However, self-control was related to age (*r* = 0.21, *p* < 0.01) and tenure (*r* = 0.29, *p* < 0.001).

**Table 3:** Descriptive statistics and correlations of Study 2.

**Variable**	***Mean***	***SD***	**1**	**2**	**3**	**4**	**5**	**6**	**7**	**8**	**9**
1. Gender	1.38	0.49									
2. Age	2.37	0.88	0.03								
3. Tenure	3.06	1.01	−0.09	0.77^***^							
4. LP based on manipulation	0.49	0.50	−0.20^**^	0.03	0.05						
5. LP based on manipulation check	3.14	1.12	−0.15^*^	0.08	0.07	0.92^***^	**(0.96)**				
6. Psychological distance	2.36	0.96	−0.06	0.00	−0.06	0.26^***^	0.30^***^	**(0.96)**			
7. Self–control	3.94	0.63	−0.05	0.21^**^	0.29^***^	0.18^*^	0.13	−0.26^***^	**(0.90)**		
8. IND_SC	3.94	0.41	−0.02	0.14	0.26^***^	0.09	0.10	0.06	0.42^***^	**(0.70)**	
9. Abusive supervision	1.80	0.63	−0.06	0.00	−0.06	0.05	0.05	0.21^**^	−0.26^***^	−0.24^**^	**(0.82)**

*N = 180. Bold value means reliability estimates (coefficient alpha) are on the diagonal*;

* *p < 0.05*,

** *p < 0.01, and*

*** *p < 0.001*.

#### Hypotheses Testing

Table 4 summarizes the regression results. Power was not related to abusive supervision in this study (M5: *b* = −0.05, *n.s*.). Thus, Hypothesis 1 was supported. Power was positively associated with psychological distance (M1: *b* = 0.49, *p* < 0.01), while psychological distance was not associated with abusive supervision (M6: *b* = 0.08, *n.s*.). However, psychological distance played a mediating role in the relationship between power and abusive supervision if self-control was not considered [indirect effect *b* = 0.06, 95% CI = (0.02, 0.14)]. The data were collected using a common method, and the number of valid samples was small. Therefore, the conclusion that Hypothesis 2 was supported can be accepted.

**Table 4:** Regression results of Study 2.

**Variable**	**Psychological distance**		**Self–control**		**Abusive supervision**		
	**M1**	**M2**	**M3**	**M4**	**M5**	**M6**	**M7**
Intercept	2.38	1.60	3.28	1.43	2.01	2.60	3.31
Gender	−0.06	−0.07	0.02	0.00	−0.09	−0.08	−0.07
Age	0.13	0.06	−0.03	0.04	0.08	0.07	0.05
Tenure	−0.16	−0.13	0.19^**^	0.09	−0.10	−0.04	−0.01
LP	0.49^**^	0.47^***^	0.21^*^	0.17^*^	0.05	0.06	0.04
IND_SC		0.22		0.52^***^			−0.31^*^
LP^*^ IND_SC		1.85^***^		−0.48^*^			−0.35
Psychological distance						0.08	0.14^*^
Self-control						−0.24^**^	−0.16
*R^2^*	0.08	0.24	0.11	0.25	0.02	0.10	0.14
Δ*R*^2^		0.16^***^		0.14^***^		0.08^**^	0.04^*^
*F*	3.73^**^	8.91^***^	5.46^***^	9.66^***^	0.67	3.10^**^	3.40^**^

*N = 180*;

* *p < 0.05*,

** *p < 0.01, and*

*** *p < 0.001*.

As seen in M3, power was positively related to self-control in this study (*b* = 0.21, *p* < 0.05); self-control was also positively associated with abusive supervision (M6: *b* = −0.24, *p* < 0.01). Hence, self-control played a mediating role in the relationship between power and abusive supervision [indirect effect *b* = −0.06, 95% CI = (−0.17, −0.01)]. Hence, Hypothesis 3 was supported.

**Figure 4:**
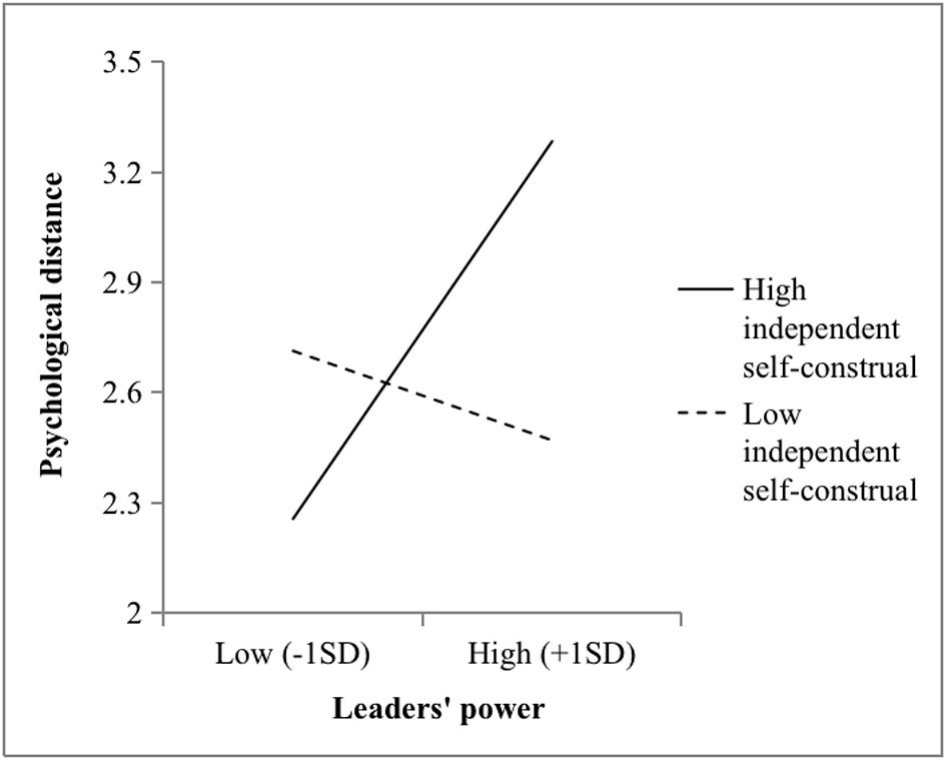
Two-way Interaction effect between the power and independent self-construal of the leaders on psychological distance in Study 2.

**Figure 5:**
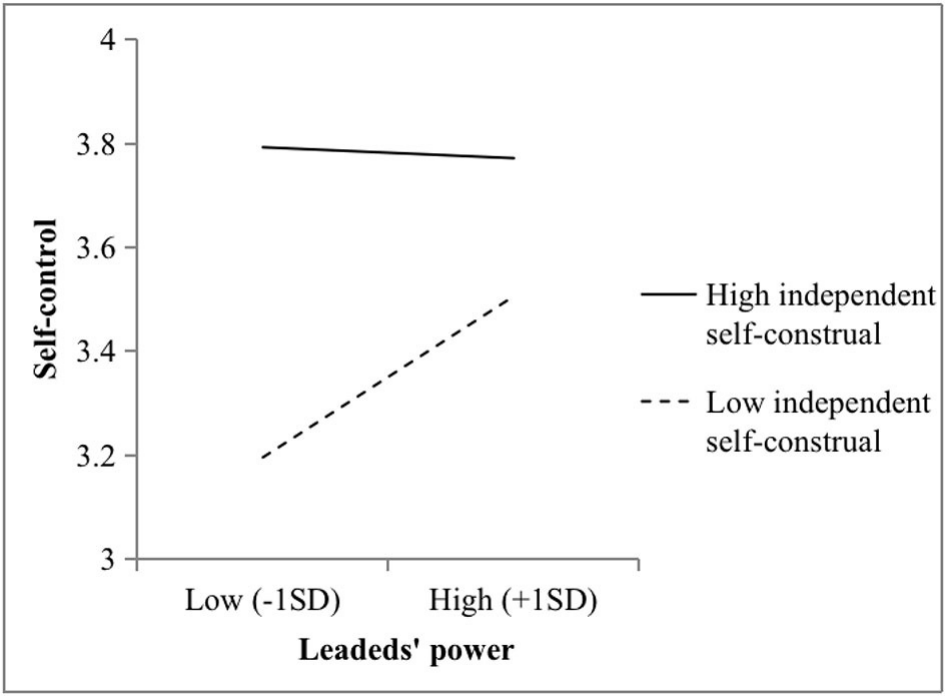
Two-way interaction effect between the power and independent self-construals of leaders on self-control in Study 2.

Next, M2 showed that power and independent self-construal interacted to predict psychological distance (*b* = 1.85, *p* < 0.001). As illustrated in Figure 4, the positive effect of power and psychological distance was significant among the supervisors with higher independent self-construals (*b* = 1.24, *p* < 0.001), while the relationship was not significant (*b* = −0.29, *n.s*.) when these independent self-construals were lower. Also, M4 showed that independent self-construals moderated the relationship between power and self-control (*b* = −0.48, *p* < 0.05). As illustrated in Figure 5, the positive effects of power and self-construal were stronger among the supervisors with lower independent self-construals (*b* = 0.37, *p* < 0.05) vs. higher independent self-construals (*b* = −0.03, *n.s*.). Thus, Hypotheses 4 and 5 were supported.

Hypotheses 6 and 7 posited that the moderating effect of the independent self-construals of leaders on the indirect effect of psychological distance and self-control was significant, respectively. Specifically, the indirect effect *via* psychological distance was more pronounced when the independent self-construals were higher (*b* = 0.21, *p* < 0.001, 95% CI = (0.09, 0.37)] rather than lower (*b* = −0.05, *p* < 0.01, 95% CI = (−0.13, −0.01)]. Furthermore, the difference between these two indirect effects was also significant [*b* = 0.26, *p* < 0.001, 95% CI = (0.11, 0.47)]. Thus, this finding meant that the moderating effect of independent self-construals on the indirect relationship between the power of leaders and abusive supervision *via* psychological distance was significant, supporting Hypothesis 6. Again, the indirect effect *via* self-control was more pronounced when independent self-construals were lower [*b* = −0.09, *p* < 0.001, 95% CI = (−0.21, −0.02)] rather than higher [*b* =0.01, *n.s*., 95% CI = (−0.07, 0.07)]. The difference between these two indirect effects was also significant [*b* = 0.09, *p* < 0.01, 95% CI = (0.02, 0.23)]. Thus, this finding meant that the moderating effect of independent self-construals on the indirect relationship between the power of leaders and the abusive supervision *via* self-control was significant, thereby supporting Hypothesis 7.

## General Discussion

This study examined the reasons why leaders abuse subordinates from the power perspective. The general answer was that they have higher power. However, not all high-power leaders abuse their subordinates. This study explored the internal mechanisms in the relationship between power and abusive supervision based on the social distance theory of power (Magee and Smith, 2013). Specifically, this study attempted to explain the internal reasons for abusive behavior from the perspective of the dual role of power. On the one hand, high power brings higher self-control, thus reducing the occurrence of management through abusive methods. On the other hand, high power also brings higher psychological distance, thus increasing abusive supervision. However, the strengths of both sides are regulated by the level of the independent self-construction of leaders.

### Theoretical Implications

The findings of this study have at least three theoretical implications. First, this study extended the current research on abusive supervision, specifically by shedding light on divergent perspectives regarding the effects of power on abusive supervision. A relevant theory suggests that the power of a manager induces them to abuse their subordinates (Lam and Xu, 2019), while other studies have confirmed that not all leaders engage in abusive management (Wee et al., 2017). Thus, the strong inference of the studies conducted in this research has taken a step toward reconciling these divergent findings by discussing the relationship between the power of leaders and their abusive supervision based on the social distance theory of power. This approach also provided a new way to resolve differences in the field of the power and abusive management of leaders. Meanwhile, this study confirmed the social distance theory of power and deepened the understanding of the role of power, that is, different directions and mechanisms of power may bring opposite effects.

Second, the exploration of the underlying mechanisms between power and abusive supervision contributed to the understanding of the antecedent of abusive leadership. To date, existing literature has mainly drawn from the perspectives of social learning, identity threat, and self-regulation impairment to explore why supervisors abuse subordinates (Tepper et al., 2017). However, these perspectives have overlooked the basis of abusive supervision, namely, that leaders have power over employees. To better explain this relationship, this study put forward two competing hypotheses and empirically verified the internal mechanism of the study from two perspectives (psychological distance and self-control). Specifically, by focusing on the perspectives of psychological distance and self-control, this study demonstrated that theoretical value can be added by shedding light on the double-edged nature of power with regard to abusive leadership. Furthermore, while previous research has largely focused on the negative effects of power on abusive supervision (Mooijman et al., 2015; Liang et al., 2016) and further zooming into the differential effects of psychological distance and self-control, the results of this study indicated that psychological distance induces managers to abuse their subordinators, whereas self-control prevents managers from treating their subordinators abusively.

Third, based on the interpersonal perspective, this study identified new boundary conditions for the well-established relationship between power and abusive supervision. These conditions can better explain the process and impact of the competitive mechanism of power on abusive management. Although the social distance theory of power has been uniformly accepted, the boundary conditions of power on outcomes (psychological distance and self-control) have not been widely discussed. In addition, the theory cannot explain the different effects of power on abusive management. Thus, this study explored supervisor-related factors, namely, independent self-construal, which can go some way toward explaining why some leaders are more prone to abuse their subordinators. In addition, this study provided new boundary conditions for the occurrence of abusive supervision. Finally, the conclusion of this study was conducive to deepening the understanding of the internal mechanisms of the role of power and the nature of the relationship between power and abusive management.

### Practical Implications

In addition to these conceptual benefits, this work study entailed important practical implications. First, this research found that the power of leaders does not directly induce abusive supervision, which occurs through psychological distance and self-control. Thus, leaders need to strive to improve their levels of self-control and control their negative emotions and impulsive behaviors in work situations as these will further reduce the occurrence of abusive behavior. Meanwhile, leaders should shorten the psychological distance between themselves and other employees (especially subordinates), which is another effective way to reduce the frequency of abusive supervision. For example, leaders could bring employees into their more intimate social spheres or put them in the inner circle of moral expansion (Crimston et al., 2016), that is, the leaders can demonstrate that they are more concerned about the feelings of their employees. Furthermore, organizations can also strengthen the self-control of leaders by making them happier (Diestel et al., 2015). The psychological distance between employees and leaders could be shortened through more league-building, family-day, and team-building activities. Secondly, abusive supervision can be reduced by promoting the negative mediating effect of self-control and weakening the positive mediating effect of psychological distance; this is the leadership trait of highly independent self-construals. In addition to the routine leadership and big five personality tests, the self-construction level scale can be used to select individuals with highly independent self-construal characteristics to undertake management work. If leaders in high positions cannot or do not care more about the feelings and interests of their employees, they will use their high power to engage in more corrupt and self-interested behaviors (Bendahan et al., 2012; Sanders et al., 2015) and subsequently engage in more abusive behaviors. Therefore, senior leaders must have low levels of independent self-construal.

### Limitations and Directions for Future Research

This research has several limitations. First, subordinates can perceive abusive supervision from multiple sources (Tepper, 2000; Zhang and Bednall, 2016). Though the power of a leader can be a major source of abusive supervision, employees may perceive this mistreatment behavior from supervisors due to other factors (e.g., Hoobler and Brass, 2006; Zhang and Bednall, 2016; Khan et al., 2017). Thus, when evaluating perceived abusive supervision, in addition to the power of leaders, subordinates may also consider other sources for clues. Future studies should test and replicate the model used in this study and integrate other sources with the power of the supervisors to predict abusive supervision. Second, the data used in this study were only collected in China, which may elicit some concerns about the generalizability of our findings with regard to different cultures. However, numerous studies on abusive supervision have been conducted in China (e.g., Naeem et al., 2019; Shen et al., 2019). Effect sizes from these studies have been found to lie within the same confidence intervals as Western samples across multiple focal outcome variables (Zhang and Liao, 2015; Mackey et al., 2017). Nevertheless, it would be valuable for future studies to examine how power relates to abusive supervision in other populations. Third, data were only collected from enterprises in the field study, without concerning other organizational forms, such as public institutions. As those sectors operate differently (Bai et al., 2001), the findings from pubic institutions may vary. In the future, studies could consider the impact of organizational forms on abusive supervision. Fourth, in the field study, the number of invalid samples was one-fifth of the total sample. Those invalid samples may have indicated less consideration of the feelings of others and less control over behaviors. Thus, the data from the invalid samples could be an important indicator. Future studies should maximize the amount of valid data to improve the reliability and validity of the conclusions.

## Data Availability Statement

The raw data supporting the conclusions of this article will be made available by the authors, without undue reservation.

## Ethics Statement

The studies involving human participants were reviewed and approved by the Ethics Committee of Shanghai University of Finance and Economics. The patients/participants provided their written informed consent to participate in this study.

## Author Contributions

CH conceived this study, designed questionnaires, collected data, wrote introduction and discussion, and finalized the manuscript for submission. ST analyzed data and wrote method and result sections. All authors contributed to the article and approved the submitted version.

## Conflict of Interest

The authors declare that the research was conducted in the absence of any commercial or financial relationships that could be construed as a potential conflict of interest.

## Publisher's Note

All claims expressed in this article are solely those of the authors and do not necessarily represent those of their affiliated organizations, or those of the publisher, the editors and the reviewers. Any product that may be evaluated in this article, or claim that may be made by its manufacturer, is not guaranteed or endorsed by the publisher.
